# Assessment of the Sensitivity of a Smartphone App to Assist Patients in the Identification of Stroke and Myocardial Infarction: Cross-Sectional Study

**DOI:** 10.2196/60465

**Published:** 2025-03-03

**Authors:** Amar Dhand, Rama Mangipudi, Anubodh S Varshney, Jonathan R Crowe, Andria L Ford, Nancy K Sweitzer, Min Shin, Samuel Tate, Haissam Haddad, Michael E Kelly, James Muller, Jay S Shavadia

**Affiliations:** 1 Department of Neurology, Brigham and Women's Hospital, Harvard Medical School, 65 Landsdowne Street, Cambridge, MA, 02139, United States, 1 617 732 5330; 2 Network Science Institute, Northeastern University, Boston, MA, United States; 3 Division of Cardiology, Department of Medicine, Unversity of Saskatchewan, Saskatoon, SK, Canada; 4 Division of Cardiovascular Medicine, Department of Medicine, Stanford University, Palo Alto, CA, United States; 5 Department of Neurology, University of Virgina, Charlottesville, VA, United States; 6 Department of Public Health Sciences, University of Virgina, Charlottesville, VA, United States; 7 Department of Neurology, Washington University School of Medicine, St. Louis, MO, United States; 8 Division of Cardiology, Department of Medicine, Washington University School of Medicine, St. Louis, MO, United States; 9 Department of Computer Science, University of North Carolina at Charlotte, Charlotte, NC, United States; 10 Division of Neurosurgery, Department of Surgery, University of Saskatchewan, Saskatoon, SK, Canada; 11 Division of Cardiovascular Medicine, Brigham and Women's Hospital, Harvard Medical School, Boston, MA, United States

**Keywords:** time-to-treatment, digital health, patient-centered care, eHealth apps, smartphones, apps, cross-sectional study, stroke, myocardial infarction, heart attack, neurology, neurological assessment, accuracy, emergency department, finger-tapping test, hospital admission, physicians, medical records, mobile health, mhealth, symptom recognition, mobile phone

## Abstract

**Background:**

Most people do not recognize symptoms of neurological and cardiac emergencies in a timely manner. This leads to delays in hospital arrival and reduced access to therapies that can open arteries. We created a smartphone app to help patients and families evaluate if symptoms may be high risk for stroke or heart attack (myocardial infarction, MI). The ECHAS (Emergency Call for Heart Attack and Stroke) app guides users to assess their risk through evidence-based questions and a test of weakness in one arm by evaluating finger-tapping on the smartphone.

**Objective:**

This study is an initial step in the accuracy evaluation of the app focused on sensitivity. We evaluated whether the app provides appropriate triage advice for patients with known stroke or MI symptoms in the Emergency Department. We designed this study to evaluate the sensitivity of the app, since the most dangerous output of the app would be failure to recognize the need for emergency evaluation. Specificity is also important, but the consequences of low specificity are less dangerous than those of low sensitivity.

**Methods:**

In this single-center cross-sectional study, we enrolled patients presenting with symptoms of possible stroke or MI. The ECHAS app assessment consisted of a series of evidence-based questions regarding symptoms and a test of finger-tapping speed and accuracy on the phone’s screen to detect unilateral arm weakness. The primary outcome was the sensitivity of the ECHAS app in detecting the need for ED evaluation. The secondary outcome was the sensitivity of the ECHAS app in detecting the need for hospital admission. Two independent and blinded board-certified physicians reviewed the medical record and adjudicated the appropriateness of the ED visit based on a 5-point score (ground truth). Finally, we asked patients semistructured questions about the app’s ease of use, drawbacks, and benefits.

**Results:**

We enrolled 202 patients (57 with stroke and 145 with MI). The ECHAS score was strongly correlated with the ground truth appropriateness score (Spearman correlation 0.41, *P*<.001). The ECHAS app had a sensitivity of 0.98 for identifying patients in whom ED evaluation was appropriate. The app had a sensitivity of 1.0 for identifying patients who were admitted to the hospital because of their ED evaluation. Patients completed an app session in an average of 111 (SD 60) seconds for the stroke pathway and 60 (SD 33) seconds for the MI pathway. Patients reported that the app was easy to use and valuable for personal emergency situations at home.

**Conclusions:**

The ECHAS app demonstrated a high sensitivity for the detection of patients who required emergency evaluation for symptoms of stroke or MI. This study supports the need for a study of specificity of the app, and then a prospective trial of the app in patients at increased risk of MI and stroke.

## Introduction

Timely recognition and intervention in neurological and cardiac emergencies can prevent severe complications and improve survival rates. Prehospital care is emerging as a critical window for deploying innovative strategies to enhance early recognition, triage, and transport in these emergencies [1]. Prehospital care is particularly important in ischemic stroke and myocardial infarction (MI), where the time from symptom onset to treatment profoundly affects prognosis [2,3]. This is particularly true in marginalized populations, where prehospital delay is a key driver of disparities in outcomes [4,5]. Thus, addressing these delays offers broad benefits for public health, economic savings, and equity in health care [6].

The urgency in addressing delays in care for patients with stroke and MI is underscored by a wealth of literature emphasizing the “golden hour”—the initial period when medical intervention is most effective [7,8]. For stroke, thrombolytic or thrombectomy therapy within this critical window reduces the extent of brain damage and improves functional outcomes [9,10]. Similarly, for MI, early reperfusion therapy is crucial in reducing infarct size and mortality [11-13]. However, despite large-scale public health campaigns and education efforts, the majority of patients fail to recognize symptoms as life-threatening, leading to delayed presentations to the emergency department (ED) and missed opportunities for early intervention [2,14,15].

In addition to patient delay, patients often make mistakes in triage decisions. Some never seek the needed emergency evaluation, while others visit an ED for symptoms that do not require emergency evaluation [16]. The initial problem is one of sensitivity and the latter is one of specificity. For screening tests, it is important to first prioritize sensitivity before specificity. This is because not recognizing the need for emergency evaluation could lead to dangerous outcomes (eg, death or morbidity) which is more serious than overdiagnosis. We acknowledge the latter is also important for any decision support tool to be useful before final deployment [17].

**Figure 1.**
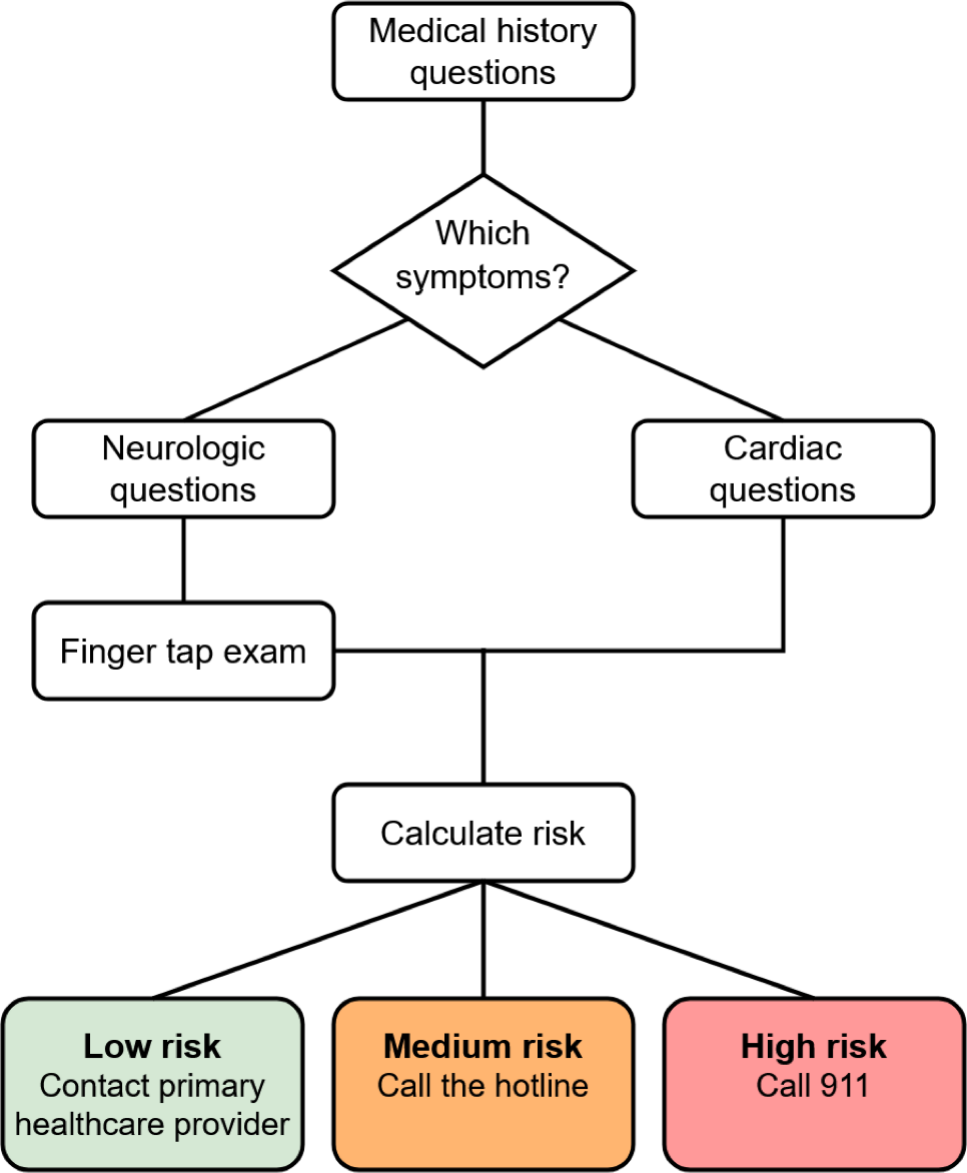
ECHAS (Emergency Call for Heart Attack and Stroke) flowchart of “history and examination” functions for symptoms concerning cardiac or stroke emergencies.

The ECHAS (Emergency Call for Heart Attack and Stroke) smartphone app is an innovative approach to bridge this gap by assisting patients in identifying symptoms of neurological and cardiac emergencies and facilitating accurate triage and a quicker intervention (Figure 1). Leveraging the ubiquity of smartphones, ECHAS aims to be a scientifically validated and regulatory-approved digital medicine technology. The app is modeled on the “history and examination” of a neurologist or cardiologist by asking a series of evidence-based questions about the user’s medical history and symptoms, as well as a finger-tapping test designed to detect unilateral weakness. Based on the user or family responses, the app calculates a risk score and recommends one of the following three actions: (1) call 911, (2) call a medical hotline, or (3) call a primary care physician now.

In the first of a series of validation steps, this study evaluated the sensitivity and usability of ECHAS in appropriately triaging patients who have already sought care in the ED for symptoms of a possible stroke, MI, or related conditions.

## Methods

### Study Design and Participants

This was a cross-sectional study at the Royal University Hospital in Saskatoon, Canada between June 2022 and July 2023. We recruited eligible patients in the ED once their initial evaluation was complete, and they were in stable condition.

The inclusion criteria for the study were patients who presented to the ED for evaluation of a possible stroke or MI. These patients were identified for exhibiting symptoms that warranted an “Stroke alert” or “MI alert” as judged by standard clinical protocol. The symptoms indicative of a stroke alert included slurred speech, asymmetric weakness or numbness, balance problems, vision changes, and headache. The symptoms that triggered an MI alert included chest discomfort, chest pressure or pain, palpitations, shortness of breath, lightheadedness or presyncope, and syncope. In addition, eligible participants were required to be 18 years or older and capable of providing informed consent.

Patients were excluded from eligibility if they were non-English speaking, had a documented history of dementia, had severe visual impairment, were unable to use a smartphone (due to physical, cognitive, or other reasons), or were unable to provide consent. A single trained research staff member enrolled all patients.

Regarding sample size, we determined the sample size a priori based on statistical and practical considerations. We estimated that a sample size of 200 patients was sufficient to provide power to detect small to medium effect sizes, ensuring robust statistical analysis of sensitivity of the app. In addition, this number was deemed feasible for a single-center study within the constraints of the available budget and study period.

The sampling method was consecutive sampling. It involved including all patients who met inclusion criteria sequentially, as they presented within a defined period, until the desired sample size was reached. This minimized selection bias. For recruitment, the research team screened eligible participants through daily chart review. We then collaborated with nurse clinicians in the ED, cardiology, and neurology units to identify potential participants. We also displayed a poster in these nursing units to assist nurses and patients to understand the study. The research team requested the bedside nurse to gauge patient interest. If patient was interested, the research team approached them at the bedside. The research team member introduced the study, obtained informed written consent, and then began collecting data.

### Ethical Considerations

We followed a protocol approved by the University of Saskatchewan Research Ethics Board (REB Bio #3380). All patients provided written informed consent (Multimedia Appendix 1) to the study. Regarding privacy, all data collected in the app was deidentified. Specifically, the responses to the questions and the finger-tapping amounts were not connected to the user. For compensation, participants did not receive any financial payment for their involvement in the study. We ensured that no individual participants may be identified in this paper.

### ECHAS Description

**Figure 2.**
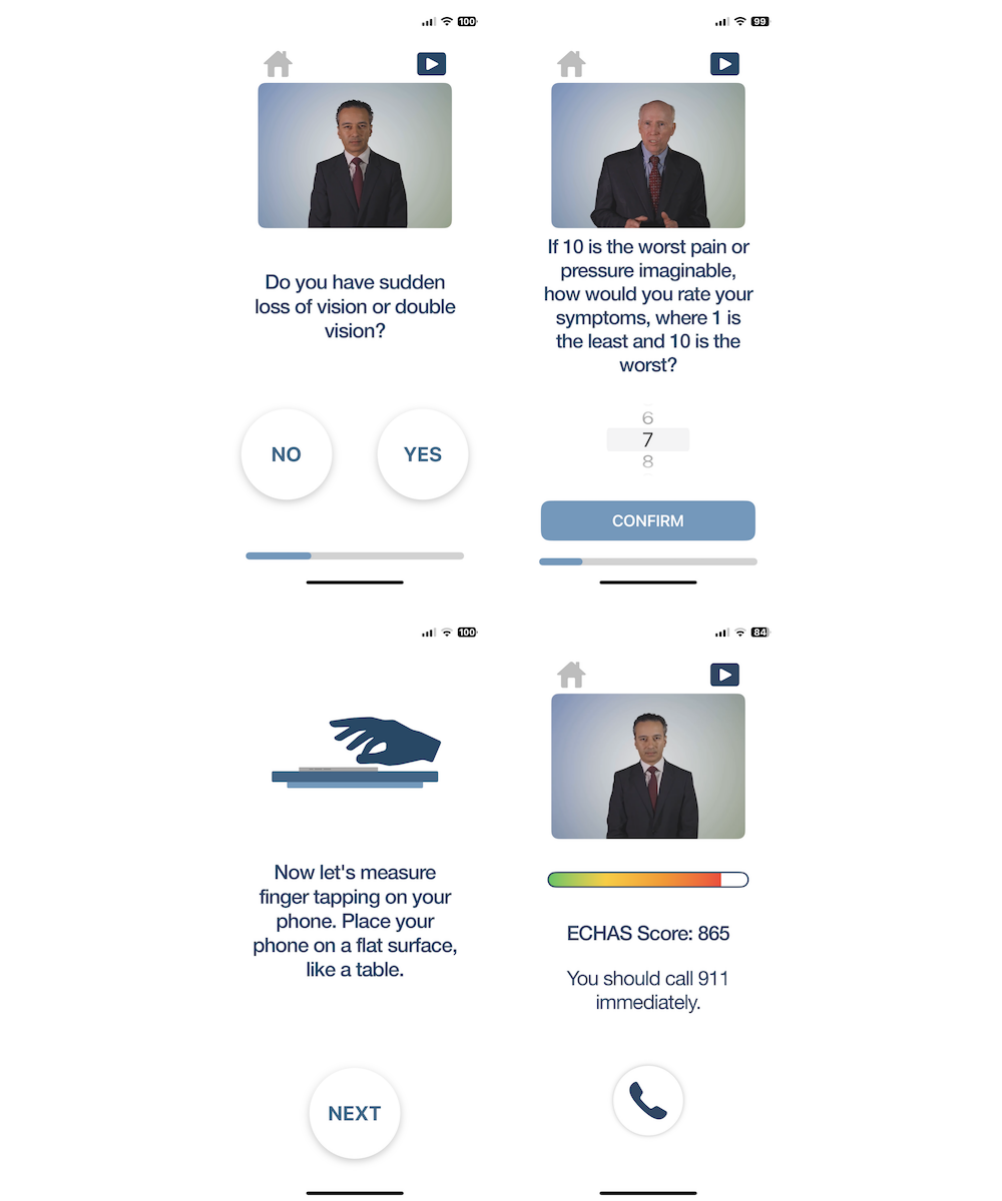
Screenshots of ECHAS (Emergency Call for Heart Attack and Stroke) including stroke question, myocardial infarction question, finger-tapping, and final suggestion. We obtained explicit consent from these individuals to use their images for publication.

All eligible patients completed a single session of ECHAS on a study-standardized Apple iPhone 13. Figure 2 displays screenshots of ECHAS. A single session included questions of symptoms and phone sensor–based examination of unilateral weakness for the stroke pathway. Participants were instructed to answer questions about symptoms based on what they experienced before coming to the ED. They answered yes or no questions about these symptoms and their previous history aligned with standard of clinical care (refer to Multimedia Appendix 2 for list of questions). Patients were asked about symptoms occurring during their decision to seek emergency evaluation that had occurred hours before engaging with the app. In many instances, the symptoms that had brought them to the ED were still present at the time of data entry in the app.

Patients in the stroke pathway additionally completed a phone sensor–based evaluation evaluating finger-tapping on the left and right hands. The app quantified the three following finger-tapping characteristics: (1) speed of finger-tapping for 10 seconds against literature-based standards, (2) comparison of finger-tapping speed on the left versus right hand, and (3) the accuracy of tapping in the middle of the target provided. Participants’ responses to questions and their finger-tapping statistics were aggregated into an ECHAS score. The ECHAS score guided one of the following three triage decisions: (1) call 911, (2) call the hot line, or (3) call your primary care physician now.

### Clinical End Points

The ground truth for the triage decision assessment was the appropriateness of the patient’s visit to the ED as judged by a retrospective review of the ED record performed by a board-certified neurologist and a board-certified cardiologist using pre-established criteria (Table 1). Each physician was blinded to the ECHAS score or triage decision and to the other adjudicator’s decision. A positive (ie, considered reasonably appropriate) ED visit was defined as a patient whose Appropriateness Scale Score was ≥3. A negative (ie, considered inappropriate) ED visit was defined as a patient whose Appropriateness Scale Score was ≤2.

**Table 1.** The Appropriateness Scale for emergency department evaluation for potential neurological and cardiac emergency.

Appropriateness Scale score		Definition
**Neurological**		
	1	No neurological imaging or neurology consultation
	2	CT^a^ head performed
	3	Neurology consultation completed
	4	Any of the following: CTA^b^ or CTV^c^ head and neck performed MRI^d^ brain performed MRA^e^ or MRV^f^ head and neck performed Invasive angiogram performed Lumbar puncture performed Admitted to observation unit or hospital admission for noncerebrovascular diagnosis
	5	Any of the following: Diagnosed with stroke, TIA^g^, ICH^h^, epidural hematoma, SDH^i^, SAH^j^ Admitted to hospital for a cerebrovascular diagnosis
**Cardiac**		
	1	No ECG^k^ taken nor troponin value measured
	2	ECG taken or single troponin value measured
	3	ECG taken, ≥2 troponin values measured
	4	ECG taken, ≥2 troponin values measured PLUS: Coronary CTA performed TTE^l^ performed Stress test performed Invasive coronary angiography performed OR (no ECG or troponin required) Arrhythmia requiring cardioversion Admission to observation unit or hospital admission for a noncardiovascular diagnosis
	5	Any of the following: Diagnosed with ST-segment elevation MI^m^ (STEMI), Non-ST elevation-acute coronary syndrome (NSTE-ACS), acute PE^n^, acute aortic dissection, cardiac arrest, or cardiogenic shock Admitted to hospital for a cardiovascular diagnosis

a CT: computed tomography.

b CTA: computed tomography angiography.

c CTV: computed tomography venography.

d MRI: magnetic resonance imaging.

e MRA: magnetic resonance angiography.

f MRV: magnetic resonance venography.

g TIA: transitory ischemic attack.

h ICH: intracerebral hemorrhage.

i SDH: subdural hematoma.

j SAH: subarachnoid hemorrhage.

k ECG: electrocardiogram.

l TTE: transthoracic echocardiography.

m MI: myocardial infarction.

n PE: pulmonary embolism.

The primary outcome was the sensitivity of the ECHAS app in detecting patients who needed ED evaluation. We compared the 2 highest acuity triage decisions of the app (call 911 or call the hotline) versus a positive classification (Appropriateness Scale Score ≥3) by the blinded physician adjudicators. We also calculated the sensitivity of ECHAS app to predict admission to the hospital for workup (Appropriateness Scale Score cutoff of 5 as positive and ≤4 as negative).

In the usability study, we determined the time to complete the ECHAS assessment and recorded the feedback of patients on the usability of the app. We recorded patient’s feedback both in the form of 2-choice or 5-choice Likert scales to measure opinions and semistructured interview responses.

### Statistical Analysis

We first completed descriptive analyses of the ECHAS score and the Appropriateness Scale Score. Then, we assessed their relationship using scatterplots, Spearman’s correlation, and unadjusted linear regression. Finally, we created 2×2 tables of ECHAS versus the ground truth and calculated the sensitivity. The study was focused on sensitivity since failure to identify emergency cases is the most dangerous potential outcome of use of the app. The study population in this ED-based context had a high prevalence of serious neurological or cardiac conditions warranting ED evaluation. Specificity will be evaluated in a subsequent study in patients who did not seek ED evaluation.

For secondary outcomes, we calculated the mean and median time that patients took to complete the ECHAS session. We created bar plots of patients’ responses on usability. Finally, we completed a qualitative analysis of the participants’ interview responses. As the qualitative analysis was ancillary to the quantitative work, we followed a “small q qualitative research” approach [18]. Using single-participant interviews with fully structured, open-ended questions, we elicited brief responses, often single sentences. We coded for patterns to describe and summarize topics and categories [19]. Throughout the process, we engaged in reflexivity by discussing patterns as a group before, during, and after coding. These discussions ensured the confirmability, dependability, credibility, and transferability of our findings.

We used the STROBE (Strengthening the Reporting of Observational Studies in Epidemiology) Statement Guideline in reporting results (Checklist 1).

## Results

### Cohort Characteristics

The cohort examined in this study consisted of 202 enrolled participants, of which 200 had ECHAS scores available. In 2 patients, the scores were not recorded due to technical error. The distribution of patients by clinical presentation included 57 patients with stroke and 145 patients with MI. The overall sex distribution was 76 women and 126 men. The median age of the participants was 62 (IQR 51-67) years. Racial composition of the cohort comprised White (n=179), Asian (n=10), Black or African American (n=1), and Indigenous (n=12). In Table 2, we present the clinical characteristics stratified by illness including baseline medical, ED presentation, stroke, and MI data.

**Table 2.** Clinical characteristics of 202 study participants from Royal University Hospital emergency department in 2022‐2023.

Variables			Presented to ED^a^ for symptoms	
			Stroke (n=57)	MI^b^ (n=145)
**Baseline medical data**				
	**Sex, n (%)**			
		Male	25 (44)	102 (70)
		Female	32 (56)	44 (30)
	Weight (kg), median (IQR)		86.0 (75.0-97.5)	90.0 (76.0-102.0)
	Height (cm), median (IQR)		168.0 (163.0-175.0)	173.5 (165.0-180.3)
	**Race, n (%)**			
		American Indian or Alaska Native	0 (0)	0 (0)
		Asian	0 (0)	10 (7)
		Black or African American	1 (1)	0 (0)
		Native Hawaiian or Other Pacific Islander	0 (0)	0 (0)
		White	53 (93)	127 (87)
		Other	3 (5)	9 (6)
	**Cigarette smoking, n (%)**			
		Current (within last 30 days)	19 (33)	41 (28)
		Never	34 (60)	72 (50)
		Quit ≥1 month	4 (7)	32 (22)
	Diabetes mellitus		9 (16)	30 (21)
	**Diabetes type, n (%)**			
		Type 1	1 (11)	2 (7)
		Type 2	8 (89)	28 (93)
	Medications taken to control blood sugars		9 (100)	23 (77)
	**Diabetes drug type, n (%)**			
		Oral medication	7 (12)	20 (14)
		GLP-1^c^ analogues	1 (2)	2 (1)
		Insulin	3 (5)	10 (7)
	Dyslipidemia requiring treatment, n (%)		16 (28)	43 (29)
	Hypertension requiring treatment, n (%)		19 (33)	65 (45)
	Previous MIs, n (%)		2 (4)	26 (18)
	Previous intervention within 30 days, n (%)		0 (0)	2 (1)
	**Type of intervention, n (%)**			
		CABG^d^	0 (0)	0 (0)
		PCI^e^ or stenting	0 (0)	2 (1)
		Hear ablation	0 (0)	0 (0)
		Heart valve surgery	0 (0)	0 (0)
	Acute coronary syndrome, n (%)		3 (5)	28 (19)
	Previous stroke, n (%)		6 (11)	7 (5)
	Previous transient ischemic attack (TIA), n (%)		7 (12)	3 (2)
**ED presentation data**				
	**Hours from symptom onset to ED arrival, n (%)**			
		Less than 3 hours	24 (43)	58 (40)
		3 to 6 hours	5 (9)	18 (12)
		More than 6 hours	27 (48)	69 (46)
	Time from symptom onset to arrival, median (IQR)		340 (113-1140)	276 (85-2880)
	**Mode of transportation to ED, n (%)**			
		EMS^f^ from home or residence	13 (23)	29 (20)
		EMS from rehab or other health care facility	0 (0)	1 (0.7)
		Personal auto	39 (68)	110 (76)
		Taxi	0 (0)	0 (0)
		Transfer from other hospital	4 (7)	5 (3)
		Other	1 (2)	0 (0)
**Stroke data**				
	**Stroke severity by NIH^g^ Stroke Scale, n (%)**			
		Mild (0‐4)	45 (79)	—^h^
		Moderate (5-14)	11 (19)	—
		Severe (15-42)	1 (2)	—
	**Finger-tapping assessment, n (%)**			
		Both sides are normal speed and equal	39 (75)	—
		Right side is slow compared with left	6 (12)	—
		Left side is slow compared with right	7 (14)	—
		Both sides are slow	0 (0)	—
	**CT^i^ results, n (%)**			
		CT not done	10 (18)	—
		Normal	30 (53)	—
		Abnormal	17 (30)	—
	**MRI^j^ results, n (%)**			
		MRI not done	36 (63)	—
		Normal	9 (16)	—
		Abnormal	12 (21)	—
	**Source document to be submitted, n (%)**			
		CT Report	46 (81)	—
		MRI Report	20 (35)	—
	TPA given, n (%)		2 (4)	—
	Mechanical thrombectomy completed, n (%)		1 (2)	—
**MI data**				
	**Cardiac biomarkers, n (%)**			
		Troponin T	—	13 (9)
		Troponin I	—	17 (12)
		High sensitive troponin T	—	130 (89)
		Creatine kinase	—	121 (83)
		Not performed	—	11 (8)
	Troponins test 1 result (ng/mL), median (IQR)		—	0.016 (0.007-0.057)
	Troponins test 1 upper reference range, median (IQR)		—	0.014 (0.014-0.050)
	Troponins test 2 result (ng/mL), median (IQR)		—	0.063 (0.016-0.711)
	Troponins test 2 upper reference range, median (IQR)		—	0.014 (0.014-0.050)
	Troponins test 3result (ng/mL), median (IQR)		—	0.083 (0.033-1.114)
	Troponins test 3 upper reference range, median (IQR)		—	0.014 (0.014-0.050)
	Creatine kinase result (U/L), median (IQR)		—	159.0 (94.0-1010.0)
	**ECG^k^ changes, n (%)**			
		Normal ECG during the event	—	49 (34)
		Abnormal	—	35 (24)
		ECG showed signs of new MI	—	34 (24)
		ECG showed signs of possible new MI	—	19 (13)
		ECG showed non-specific signs	—	7 (5)
	Cardiac catheterization performed, n (%)		—	77 (53)
	Coronary imaging performed, n (%)		—	12 (8)
	Stress echo ordered, n (%)		—	3 (2)
	New cardiac medications started, n (%)		—	80 (55)
	**Class of new cardiac medicines prescribed, n (%)**			
		Antiplatelet	—	76 (52)
		Anticoagulation	—	74 (51)
		Lipid-lowering	—	30 (21)
		Antihypertensive	—	24 (16)

a ED: emergency department.

b MI: myocardial infarction.

c GLP-1: Glucagon-like peptide-1.

d CABG: coronary artery bypass graft.

e PCI: percutaneous coronary intervention.

f EMS: emergency medical services.

g NIH: National Institutes of Health.

h Not applicable.

i CT: computed tomography.

j MRI: magnetic resonance imaging.

k ECG: electrocardiogram.

### ECHAS Score Versus Appropriateness Scale Scores

**Figure 3.**
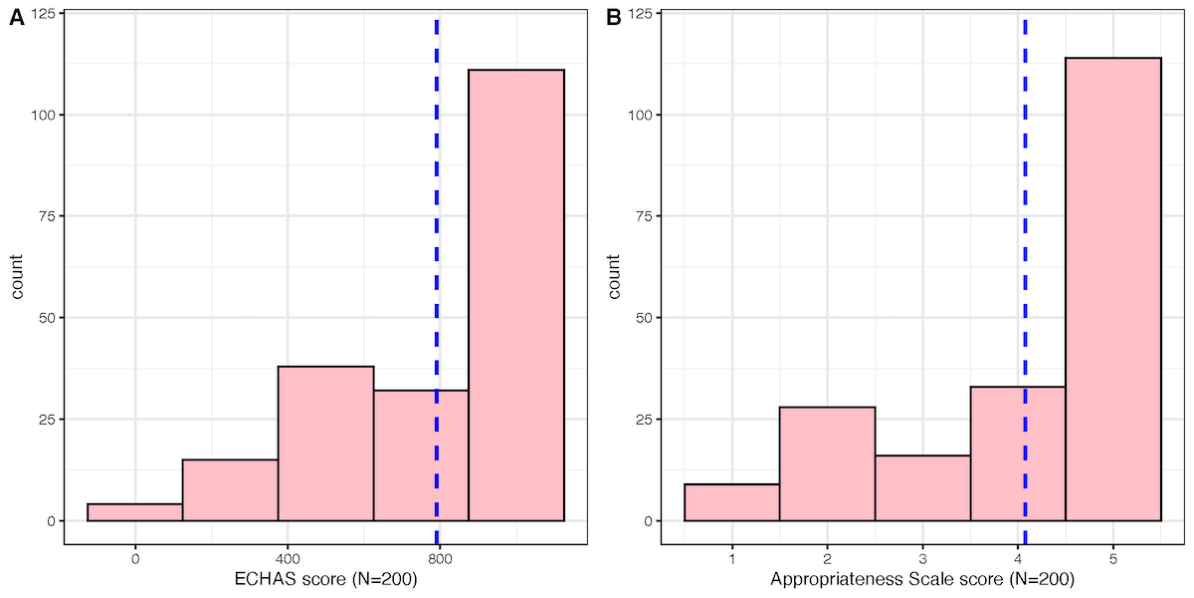
Distribution of the ECHAS (Emergency Call for Heart Attack and Stroke) score and appropriateness scale score (ground truth) with the median indicated by the blue line in 202 study participants.

The distribution of scores for the ECHAS score and the Appropriateness Scale were similar, as illustrated in Figure 3. This similarity suggests alignment in the measurement of clinical outcomes by these 2 scales.

We observed a significant correlation between the ECHAS score and the Appropriateness Scale Score, with a Spearman correlation coefficient 0.41 (*P*<.001). This pattern of correlation was consistent when stratified for patients in both the MI and stroke pathways. This association was confirmed in a simple linear regression, where the ECHAS β-coefficient was determined to be 0.002 (SE=0.003, *P*<.01), with a constant of 2.557.

The agreement of ECHAS scores with the ground truth, as adjudicated by 2 independent physicians, is presented in Table 3. This 2×2 table shows that the physicians adjudicated the cases had 97% agreement, suggesting low interobserver variability.

**Table 3.** Two-by-two tables of the ECHAS (Emergency Call for Heart Attack and Stroke) app triage advice versus adjudicators’ determination of appropriateness of emergency department visit.

ECHAS app		Positive	Negative	Sensitivity	Specificity
**Adjudicator 1**				0.98	0.08
	Positive	159	34		
	Negative	4	3		
**Adjudicator 2**				0.97	0.07
	Positive	153	40		
	Negative	4	3		

The average sensitivity of ECHAS for appropriate triage was 0.98. The sensitivity for identifying patients admitted to the hospital for possible stroke or MI was 1.0, indicating that ECHAS detected all patients who were highest acuity. The average specificity of ECHAS for appropriate triage was 0.08.

### Secondary Outcomes

The average time to complete the assessment using ECHAS varied by 111 (SD 60) seconds for stroke and 60 (SD 33) seconds for MI.

**Figure 4.**
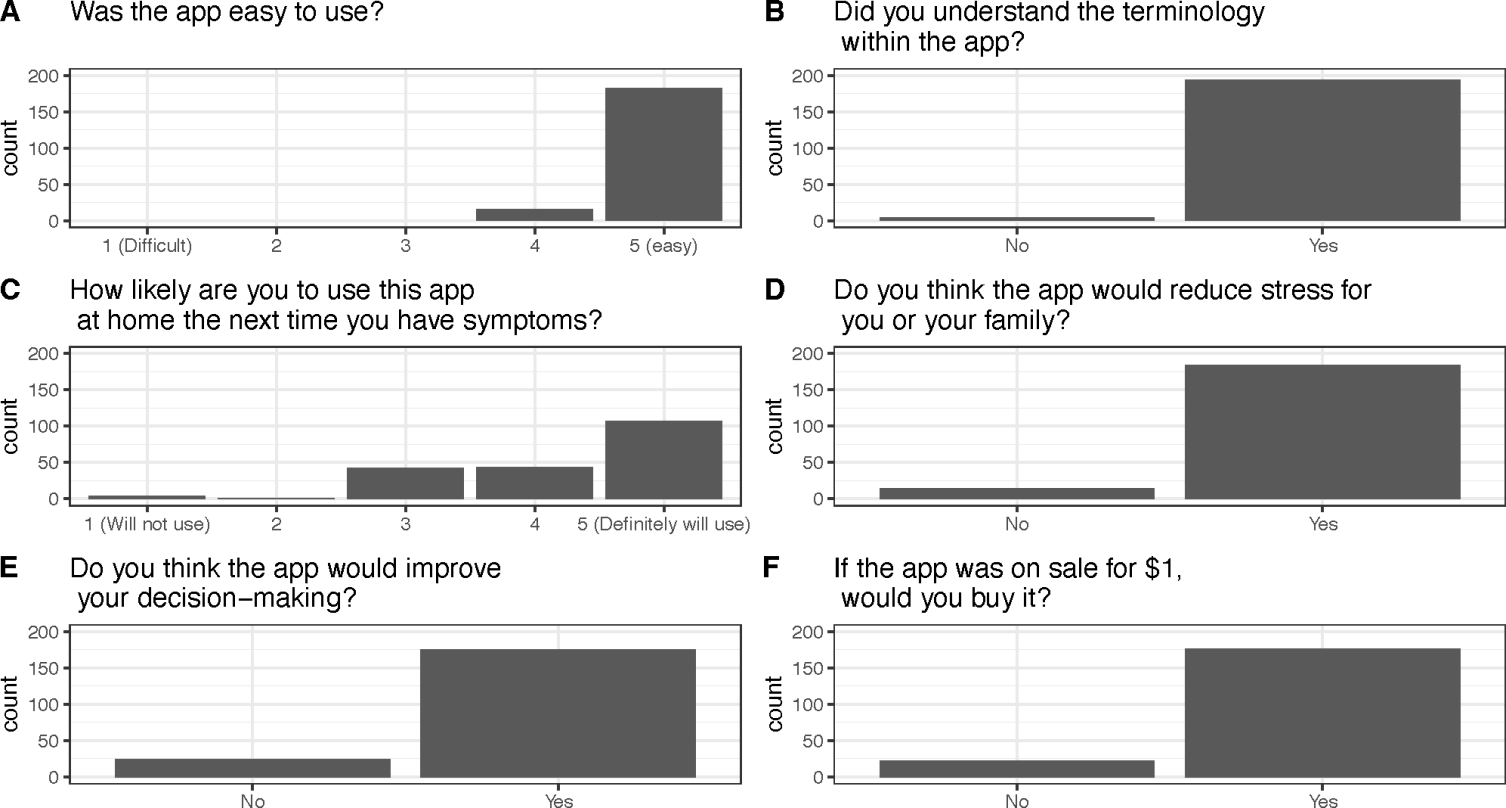
Usability of the ECHAS (Emergency Call for Heart Attack and Stroke) app according to study participants.

Patients reported finding ECHAS usable, understandable, and worthwhile in acute emergencies at home, as shown in Figure 4. Specific comments from patients, which provide insights into their experiences and perceptions of the app, are detailed in Textbox 1.

Textbox 1. **Patients’ experiences and perceptions of the ECHAS (Emergency Call for Heart Attack and Stroke) after use in the Royal University Hospital emergency department.**

**App is helpful, useful, and positive**

- *Good app. Very useful for delayers. Could save lives.*
- *I wish I had this app in my mobile 2 days back.*
- *The next time I experience any symptoms that I don’t understand, I would use the app to help me make the correct decision.*
- *I would inform my colleagues that this app is available. I would use it myself if I was having chest pain again.*
- *Consider if vitals measurement could be done by the smart phone.*
- *If I have symptoms again I would use again.*

**App as important within family networks**

- *I would definitely buy the app for my family members.*
- *If the app could get the summary of all the questions answered at the end (like a PDF copy)- may be beneficial for EMS/ER to look at the responses to reduce the time for reevaluation or asking same questions.*
- *I would use the app whenever I thinks my father is having a possible heart attack or stroke.* [The patient’s daughter]

**Suggestions for improvement:**

- *Don’t know what to select. I have both severe headache and chest pain. Thought could be either of stroke or heart attack.*
- *Chest pain felt like a prior heart attack- so called 911. I would call 911 irrespective of the app.*
- *Don’t remember my blood pressure.*
- *Not sure if I am having heart attack or stroke which one to select to begin.*

## Discussion

### Principal Results

In this initial validation study, we evaluated the sensitivity and usability of the ECHAS smartphone app in 202 patients presenting to an ED for symptoms of a possible stroke or MI. We found a significant correlation between ECHAS scores and the Appropriateness Scale scores, indicating the app was successful in identifying patients who should seek ED evaluation. We found that ECHAS demonstrated high sensitivity of 0.98, especially in identifying high-acuity stroke or MI patients, with a unanimous detection rate for those patients admitted. In addition, the app proved to be efficient, requiring on average 111 (SD 60) seconds for stroke, and 60 (SD 33) seconds for MI. Patient feedback highlighted the potential usability and effectiveness of ECHAS in emergency situations at home.

These findings support continued development of the app which requires study of specificity and a prospective study. The specificity in this study of patients who were already on the MI or Stroke Alert pathway in the emergency room was low (0.08). This means that ECHAS did not detect patients who did not need more comprehensive cardiac workup (ECG taken, ≥2 troponin values measured or more advanced studies) or neurological workup (neurology consultation and advanced imaging). The low specificity could mean that the app would over-recommend ED visits leading to false positives. However, it is unknown how ECHAS would perform in otherwise healthy persons at home. This needs further prospective study. In this study, we deliberately prioritized sensitivity because the consequences of low sensitivity are more dangerous than those of low specificity.

### Comparison With Previous Work

The development of ECHAS is informed by a communication and health behavior theory suggesting that mobile health technologies address constraints of space and time that hinder more traditional forms of mass media communication [20]. Specifically, mobile health tools have shown promise to enhance self-management, adherence to treatment, and health literacy among patients with chronic conditions [21]. Furthermore, certain mobile health solutions provide an opportunity for real-time monitoring and assessment, which is particularly valuable in emergency scenarios where every second counts [22]. The ECHAS study contributes to these trends in the literature by demonstrating initial accuracy of a potentially ubiquitous tool that combines a physician-informed assessment and sensor-based monitoring to empower patients and families to determine actions during a worrisome moment.

The ECHAS study adds to the evolving landscape of digital medicine technologies aimed at enhancing acute symptom detection and emergency medical system alerting [23,24]. For example, Wasselius et al [25] showed the potential of wearable accelerometers to detect unilateral arm paresis. In the Apple Heart Study, Perez et al [26] showed that a wearable algorithm could correctly identify atrial fibrillation in users who were notified of irregular pulses. Garcia et al [27] introduced a mobile app that detects key symptoms of stroke, such as smile asymmetry, speech difficulties, and arm weakness, showing effective early detection capabilities. Similarly Stroke Cognitive Medical Assistant by Khriyenko et al [28] used IBM Watson tools to help users detect face asymmetry, speech difficulties, and arm weakness. These advancements collectively underscore a significant shift toward integrating technology in the fight against acute medical emergencies.

### Study Strengths

This study’s strengths included a large sample size of patients in acute settings, the use of 2 board-certified and blinded adjudicators for end point determination, and the evaluation of ECHAS against the appropriateness of ED visits rather than just confirmed stroke or MI diagnoses. In addition, this collaborative effort between neurologists and cardiologists addresses the needs of patients with vascular risk factors who are at risk for both neurological and cardiac emergencies. Finally, these positive results were achieved with a sensor that only detects finger-tapping weakness. Future versions of ECHAS, being tested at the time of this study, include sensors for facial droop detection and slurred or incomprehensible speech detection. This should improve specificity. Together with finger-tapping weakness, ECHAS future versions would allow objective detection of face, arm, and speech abnormalities (FAST) on a smartphone.

### Limitations

As an initial investigation of ECHAS, this study had limitations. Its cross-sectional design may introduce recall bias, affecting the accuracy of reported outcomes. In addition, the performance of the app, tested in a controlled clinical environment on a standardized phone, might not mirror its usability or accuracy in real-world, at-home scenarios during worrisome moments. The study was conducted in a single center with limited diversity in terms of demographics and disabilities. The version of ECHAS evaluated in this study included sensors limited to detecting unilateral weakness through finger tapping. As stated above, ECHAS versions now being tested also include sensors for detecting facial asymmetry and speech difficulties. The study’s cohort composition limited the ability to assess specificity, which is the app’s ability to correctly identify those without the condition. This is an important metric for avoiding false positives that requires further study. We are planning future prospective studies involving at-home app deployment to address these limitations and provide a clearer understanding of ECHAS’ accuracy, specificity, and usability in real-life settings with lower risk cohorts. We also aim to study ECHAS across diverse populations to test accessibility and usability in a wide range of users.

### Conclusions

In this study of 202 patients, we found that ECHAS was highly sensitive and easily usable in guiding patients to identify medical emergencies without input by health care personnel. The findings support further research to determine specificity and a study of the app’s prospective use in home settings. Overall, this “history and examination” app adds to the burgeoning array of digital medicine tools designed to improve prehospital care, highlighting its potential to empower patients and enhance early intervention strategies.

## Supplementary material

10.2196/60465 Multimedia Appendix 1 Informed consent form.

10.2196/60465 Multimedia Appendix 2 List of ECHAS questions.

10.2196/60465 Checklist 1 STROBE (Strengthening the Reporting of Observational Studies in Epidemiology) checklist.

## Abbreviations

ECHAS: Emergency Call for Heart Attack and Stroke
ED: emergency department
MI: myocardial infarction
STROBE: Strengthening the Reporting of Observational Studies in Epidemiology
