# Supplementary material for: Assessment of the Sensitivity of a Smartphone App to Assist Patients in the Identification of Stroke and Myocardial Infarction: Cross-Sectional Study
Source: JMIR Form Res. 2025 Mar 3;9:e60465. doi: 10.2196/60465 (PMC11892415; doi:10.2196/60465)
Supplement: Multimedia Appendix 1 [file formative-v9-e60465-s001.pdf]

PARTICIPANT INFORMATION AND CONSENT FORM

STUDY TITLE: Assessment of a Prehospital Mobile App and Sensor Triage System to Detect Neurologic and Cardiac Emergencies: The ECHAS Assessment Study (ECHAS- One)

PRINCIPAL INVESTIGATOR: Dr. Jay Shavadia, Cardiologist, Division of Cardiology, Department of Medicine, University of Saskatchewan

CO-INVESTIGATORS: Dr. Michael Kelly, Dr. Amar Dhand, Dr. James Muller and Dr. Anubodh Varshney

CONTACT PHONE NUMBER: Dr. Jay Shavadia at 306-986-2260

EMAIL: jss372@mail.usask.ca

INTRODUCTION

You are invited to participate in the above-mentioned research study because you have recently had a possible medical emergency that led you to contact emergency services. Your participation is voluntary. It is up to you to decide whether or not you wish to take part. If you wish to participate, you will be asked to sign this form. If you do decide to take part in this study, you are still free to withdraw at any time and without giving any reasons for your decision.

If you do not wish to participate, you will not lose the benefit of any medical care to which you are entitled or are presently receiving. It will not affect your relationship with study doctor Dr. Jay Shavadia.

Please take the time to read the following information carefully. You can ask the researcher to explain any words or information that you do not clearly understand. You may ask as many questions as you need. Please feel free to discuss this with your family, friends, or family physician before you decide.

WHO IS CONDUCTING THIS STUDY?

This study is led by Dr. Jay Shavadia, Division of Cardiology, Department of Medicine, University of Saskatchewan. The costs of the labor for this research study are being paid by ECHAS, LLC., the company developing the app to help patients decide when to call 911 for a stroke, heart attack or related emergency.

WHY IS THIS STUDY BEING DONE?

It is often difficult for a patient to know if a heart attack or stroke is in progress, and therefore difficult to know if it is necessary to call for emergency care. A timely call is important because opening of an artery soon after the onset of symptoms can reduce the severity of a heart attack or

stroke. While many patients do not call soon enough, others call for minor symptoms that do not require emergency services.

Since most patients have access to a smartphone, it becomes possible to provide expert medical information via the phone at the time symptoms of a possible heart attack or stroke begin. ECHAS, LLC, is a private company developing an app for the smartphone that will assist patients in the difficult decision to place an Emergency Call for Heart Attack and Stroke (ECHAS). You are being asked to participate in the first test in patients of the ECHAS app to be conducted in 200 patients. The plan is to first determine how the app would have performed if you had the app prior to your recent emergency. In a second study (to be conducted after completion of ECHAS-One), the ability of the app to predict the occurrence of a heart attack or stroke will be tested.

WHAT DOES THE STUDY INVOLVE?
The study is designed to be brief and avoid any change in your routine medical care. If you consent, you will be shown the ECHAS app on a smartphone. The app will ask several background questions such as your history of heart attack or stroke, age, gender and other variables that influence the risk of a cardiovascular event. Then you will be asked to answer several other questions about your symptoms just prior to the episode which led you to call 911. After the question are completed, you will be asked to give you opinion of the usability of the app. We will also ask permission for access to your hospital record to determine the results of your emergency evaluation.

It is anticipated that the research interview will take 20 to 30 minutes of your time. There will be no blood samples or diagnostic testing for this study.

ARE THERE POSSIBLE RISKS OR DISCOMFORTS?
The study is limited to an interview of 20 to 30 minutes, and access to your medical records. There are no physical risks or discomforts.

The main risk of study participation is the inadvertent release of your personal health information. The researchers have taken measures to protect the privacy of your information and this risk is considered very small.

WHAT ARE THE BENEFITS OF PARTICIPATING IN THIS STUDY?
You are not expected to benefit directly from participating in this study. However, if you choose to participate in this study, the knowledge gained will assist in the development of this smartphone app that will help patients know when to call for emergency help with a possible heart attack or stroke.

WHAT HAPPENS IF I DECIDE TO WITHDRAW?
Your participation in this research study is voluntary. You maintain your right to withdraw from the study at any time, including research data. There will be no penalty or loss of benefits if you chose to withdraw. Your withdrawal will not affect the quality of received care or interaction with your healthcare staff.

WILL I BE INFORMED OF THE RESULTS OF THE STUDY?
A copy of the publication can be sent to you via an email or mail with the letter explaining the aggregate results. No information that discloses your identity will be released or published. We anticipate this research study to complete by Jun 2023 and the aggregate results should be known by December 2023. Alternatively, a description of this clinical trial will be available on http://www.clinicaltrials.gov (registration number NCT05230069), as required by U.S. Law. This Website will not include information that can identify you. At most, the Website will include a summary of the results. You can search this Website at any time.

WHAT WILL THE STUDY COST ME?
You will not be charged for any research-related procedures. You will not be paid for participating in this study. You will not receive any compensation, or financial benefits for being in this study, or as a result of data obtained from research conducted under this study.

WILL MY TAKING PART IN THIS STUDY BE KEPT CONFIDENTIAL?
In Saskatchewan, the Health Information Protection Act (HIPA) defines how the privacy of your personal health information must be maintained so that your privacy will be respected. The study data will be stored securely (in a locked cabinet contained within a locked office under the supervision of the Principal Investigator) by the study team for a minimum of 5 years after the results are published. Your confidentiality will be respected. A study code will be attached to your data instead of your name. Your personal identifiers will not be collected on the ECHAS app. Your clinical data will be entered into the University of Saskatchewan's research electronic data capture system called REDCap. Your identifiers will not be entered into the REDCap system. Data on the REDCap will be accessible to the research staff only after the principal investigator's approval. Research staff would need their unique username and password to log on to the REDCap. The results that will be reported will be a combination of all of the data, your individual information will not be reported. No information that discloses your identity will be released or published without your specific consent to the disclosure. Research records and medical records identifying you may be inspected by the University of Saskatchewan Biomedical Research Ethics Board in the presence of the Principal Investigator for quality assurance and monitoring purposes. However, no records which identify you will be allowed to leave the researchers' office. The research team intends to publish the results of this research in scientific journals and to present the findings at related conferences and workshops, but your identity will not be disclosed.

WHO DO I CONTACT IF I HAVE QUESTIONS ABOUT THE STUDY?
If you have any questions or desire further information about this study before or during participation, you can contact Principal Investigator Dr. Jay Shavadia at 306-986-2260.

If you have any concerns about your rights as a research participant and/or your experiences while participating in this study, contact the Chair of the University of Saskatchewan Research Ethics Board, at 306-966-2975 (out of town calls 1-888-966-2975). The Research Ethics Board is a group of individuals (scientists, physicians, ethicists, lawyers, and members of the community) that provide an independent review of human research studies. This study has been reviewed and approved on ethical grounds by the University of Saskatchewan Research Ethics Board.

CONSENT TO PARTICIPATE

STUDY TITLE: Assessment of a Prehospital Mobile App and Sensor Triage System to Detect Neurologic and Cardiac Emergencies: The ECHAS Assessment Study (ECHAS- One)

Prior to enrolment in the study, you must agree to participate in the study by signing this consent form. The signature line for the 'person obtaining consent' can be left blank and will be completed upon receipt of the signed form by one of the researchers. By signing this document, you have consented to the bulleted statements below:

- I have read the information in this consent form
- I understand the purpose and procedures and the possible risks and benefits of the study
- I was given sufficient time to think about it
- I had the opportunity to ask questions and have received satisfactory answers
- I understand that I am free to withdraw from this study at any time for any reason and the decision to stop taking part will not affect my future relationships
- I give permission to the use and disclosure of my de-identified information collected for the research purposes described in this form
- I understand by signing this document I do not waive any of my legal rights
- I will be given a signed copy of this consent form
- I understand that this study will not provide any benefits to me
- I give permission for the access of my identifiable personal health information for the research purposes described in this form

  Permission for future research:

  ☐ Yes, I permit the research team to contact me for future research (ECHAS-Two)
  ☐ No, I do not permit the research team to contact me for future research (ECHAS-Two)

I agree to participate in this study:

Printed name of the participant	Signature	Date; Time

Printed name of the person obtaining consent	Signature	Date; Time
