# Supplementary material for: Assessment of the Sensitivity of a Smartphone App to Assist Patients in the Identification of Stroke and Myocardial Infarction: Cross-Sectional Study
Source: JMIR Form Res. 2025 Mar 3;9:e60465. doi: 10.2196/60465 (PMC11892415; doi:10.2196/60465)
Supplement: Multimedia Appendix 2 [file formative-v9-e60465-s002.pdf]

SUPPLEMENT

Assessment of the Sensitivity of a Smartphone App to Assist Patients in Identification of Neurological and Cardiac Emergencies: Cross-Sectional Study

Dhand et al.

Supplement 1: List of ECHAS questions

Past Medical History questions

- What is your gender?
- What year were you born?
- What is your weight?
- What is your height?
- Have you smoked any tobacco products in the past year?
- Have you ever been diagnosed with diabetes, or do you take medications to control your sugars?
- What is your most recent systolic blood pressure (top number of the blood pressure) measurement?
- Do you take any medications to control your blood pressure?
- Have you had a heart attack in the past?
- Have you had any of the following in the past month: stenting or heart artery intervention, heart surgery, heart ablation, or heart valve surgery?
- Have you had a stroke in the past?
- Have you had a mini-stroke or TIA in the past?

Stroke questions

- (If indicated previous mini-stroke) Are your symptoms similar to your previous mini-stroke?
- (If indicated previous stroke) Are your symptoms similar to your prior stroke?
    - Are these similar symptoms new?
- Does one side of your face droop or feel numb?
- Is your speech slurred?
- Is one arm weak or numb?
- Do you have sudden loss of vision or double vision?
- Is your walking unsteady or is one of your legs weak?
- Do you have a sudden headache?
- Did you have an episode of stiffening or jerking movements?
- Are these symptoms new?
- Did your symptoms come on gradually (like over the span of 30 minutes)?
- Did you drink any alcohol in the past 6 hours?

MI questions

- Do you have pressure or pain in your chest?
    - Does the pressure or pain feel like a prior heart attack?
    - Can you feel the pressure or pain in your arms, neck, or jaw?
    - If 10 is the worst pain or pressure imaginable, how would you rate your symptoms, where 1 is the least and 10 is the worst?
    - Does the pain or pressure get worse when you press where it hurts?
    - Does the pain or pressure get worse when you move your arm?
- Have you been sweating more than usual during these symptoms?
- Do you have nausea or fatigue?

- Do you have new trouble breathing during these symptoms?
- Do you have new tearing or sharp pain in your back?
- In the hour prior to your new symptoms, did you experience: anger, emotional distress, or heavy physical exertion?
